# Ready for Transfer to Adult Care? A Triadic Evaluation of Transition Readiness in Adolescents With Congenital Heart Disease and Their Parents

**DOI:** 10.1177/1074840719864255

**Published:** 2019-07-25

**Authors:** Åsa Burström, Mariela Acuña Mora, Maria Öjmyr-Joelsson, Carina Sparud-Lundin, Annika Rydberg, Katarina Hanseus, Björn Frenckner, Margret Nisell, Philip Moons, Ewa-Lena Bratt

**Affiliations:** 1 Karolinska Institutet, Stockholm, Sweden; 2 Astrid Lindgren Children’s Hospital, Stockholm, Sweden; 3 University of Gothenburg, Sweden; 4 KU Leuven, Belgium; 5 Umeå University, Sweden; 6 Skåne University Hospital, Lund, Sweden; 7 The Red Cross University College, Stockholm, Sweden; 8 The Queen Silvia Children’s Hospital, Gothenburg, Sweden

**Keywords:** adolescents, congenital heart disease, readiness for transition, parents

## Abstract

Transfer to adult care for adolescents with chronic conditions ought to be determined by transition readiness. The aims of this study were (a) to describe the level of readiness for transition in adolescents with congenital heart disease, (b) to compare adolescents’ assessment of transition readiness with their parents’ assessments, and (c) to study potential correlates of transition readiness. A total of 157 triads of adolescents aged 14 to 18 years and their parents completed the Readiness for Transition Questionnaire. Adolescents scored higher on overall readiness than their parents. Multivariable analyses revealed that higher levels of adolescents’ overall readiness were associated with a less threatening view of the illness, a higher level of empowerment, and with higher mothers’ and fathers’ overall readiness scores. Adolescents’ responsibility scores were positively associated with age and parental adolescent responsibility scores. Parental involvement scores were negatively associated with adolescents’ age and positively with the mothers’ parental involvement scores. By using a triadic evaluation, the results of the present study significantly extend what is currently known about this population.

Today, due to improvements in medical care, an increasing number of children with congenital or childhood onset conditions survive into adulthood (American Academy of Pediatrics et al., 2011). This has led to a growing population of young persons with long-term illnesses requiring lifelong medical follow-up (Perrin, Bloom, & Gortmaker, 2007).

Regardless of their chronic condition, adolescents require well-planned and well-coordinated transitional care and sufficient knowledge about their condition to become responsible of their own care and health in adulthood (American Academy of Pediatrics et al., 2011; Rosen, Blum, Britto, Sawyer, & Siegel, 2003). The concept of health care transition is defined by Blum and colleagues (1993) as a “the purposeful, planned movement of adolescents and young adults with chronic physical and medical conditions from child-centered to adult oriented health care systems” (p. 570). Gilleland, Amaral, Mee, and Blount (2012) developed the Readiness of Transition Questionnaire (RTQ) and conceptualized readiness for transition as the adolescent’s readiness “to assume complete responsibility for their health care (e.g., process) and their readiness to transfer to adult medical care (e.g., event)” (p. 87). Furthermore, transition readiness captures the process of building the capacity of adolescents and those who are involved in his or her medical care to prepare for, enter, continue, and complete transition (Gilleland et al., 2012).

It is crucial that nurses involve both the adolescent and parents by providing information and education about the transition process and changing roles (American Academy of Pediatrics et al., 2011; Joly, 2015). Knowledge and awareness about the transition process and transfer to adult care has been shown to be associated with increased self-efficacy and self-management (van Staa, van der Stege, Jedeloo, Moll, & Hilberink, 2011). For a young person with a chronic condition, it is a challenge to gradually gain more self-management skills while trying to live as his or her healthy peers. It is also challenging for the parents to shift roles, decrease their involvement in care, and experience worries about the future and the risk of detoriation (Waldboth, Patch, Mahrer-Imhof, & Metcalfe, 2016). Parents play an important role in this process facilitating adolescents’ progress toward self-management and also supporting the child in communication with the health care providers (HCP) (Bratt, Burström, Hanseus, Rydberg, & Berghammer, 2018; Burström, Öjmyr-Joelsson, Bratt, Lundell, & Nisell, 2016; Helgeson, Reynolds, Siminerio, Escobar, & Becker, 2008). Shared responsibility and parental support improve the adolescents’ self-efficacy and are important facilitators in gaining knowledge and skills and in promoting their self-care. Furthermore, they facilitate future follow-up with uninterrupted care (Goethals et al., 2017; Goossens, Bovijn, Gewillig, Budts, & Moons, 2016; Helgeson et al., 2008). It is also important to highlight other potential sources of support. Peers become more important during adolescence (Oris et al., 2016). A recent study among oncology survivors shows that both family and friends are important in giving practical and emotional support. Furthermore, spending time with peers helps the adolescents to make autonomous decisions (McDonnell, Shuk, & Ford, 2018).

In most health care settings, chronological age is decisive for timing of transfer to adult care, as opposed to considering self-management skills, maturity, or general assessment of transition readiness. However, this is not in line with international guidelines, which advocate that timing of transfer should be guided by factors such as emotional maturity and developmental level (American Academy of Pediatrics et al., 2011).

An example of a chronic condition with increasing survival and a growing number of young persons is congenital heart disease (CHD). Many adolescents with CHD need lifelong medical follow-up (Sable et al., 2011; Silversides et al., 2010). CHD is not a uniform condition. It ranges from mild conditions with medical checkups every 3 to 5 years, to complex conditions with several future medical interventions (surgery and catheterizations) and with medical checkups several times a year (Marelli & Gurvitz, 2011). In general, transfer to adult care for adolescents with CHD is predetermined by age and, when the adolescents are 18 years old, most are transferred to adult care (Hilderson et al., 2009).

There is still a lack of knowledge about potential factors associated with transition readiness. To improve preparation for transfer to adult care, it is relevant to identify important correlates. Such information may assist nurses in developing successful family-focused interventions addressing dealing with a chronic condition throughout the life course. The aim of this study, therefore, was (a) to describe the level of readiness for transition in adolescents with CHD, (b) to compare adolescents’ assessment of transition readiness with their parents’ assessments, and (c) to study potential correlates of transition readiness in adolescents with CHD.

## Method

As part of the STEPSTONES project (Swedish Transition Effects Project Supporting Teenagers with chrONic mEdical conditionS), we conducted a cross-sectional questionnaire study.

### Setting and Participants

Data from the Swedish registry of CHD, SWEDCON (SWEDCON, 2016) were used to identify eligible patients. Inclusion criteria were adolescents aged 14 to 18 years with a CHD and in need of medical follow-up in adulthood. The participants were followed up at one of four university hospitals in Sweden (Gothenburg, Lund, Stockholm, and Umeå). Exclusion criteria included syndromes affecting cognitive abilities, heart transplantation, acquired heart diseases, illiteracy, or non-Swedish speaking. In total, 593 eligible participants were identified fulfilling the inclusion criteria. Their parents were also invited to participate.

### Data Collection

A set of questionnaires was sent to all eligible adolescents and their parents. Study information and an informed consent form were sent with the questionnaires. If no response was received, two reminders were sent by mail after 3 and 5 weeks, respectively, and with a last reminder by telephone after 7 weeks (Dillman, 1983). Ethical approval for the study was received from the Ethics Board in Gothenburg (diary number 953-13) and the study was conducted according to the Helsinki Declaration (World Medical Association, 2013).

### Variables and Measurement

For assessing the adolescents’ readiness for transition, the Readiness for Transition Questionnaire (RTQ) developed by Gilleland and colleagues (2012) was used. To compare the adolescents’ overall readiness for transition with that of their parents’ assessment, the proxy version of RTQ was filled out by the parents (Gilleland et al., 2012).

The RTQ was translated into Swedish following a standardized translation procedure using forward and backward translation, in a two-stage procedure. Four Swedish-speaking translators (four researchers with knowledge in the field) performed the forward translation independently (Wild et al., 2005). Discrepancies were resolved between their versions. Furthermore, a backward translation into English was performed by an authorized translator and native speaker of English, and further discrepancies were resolved (Wild et al., 2005). After evaluating face validity, an additional response option (not relevant) was added to the items corresponding to adolescents’ responsibility and parental involvement. This option was included as there were items related to medication and frequent contact with HCP that are not as relevant for patients with CHD. Moreover, the item related to health care insurance was removed (also removed from the RTQ-proxy version), as Swedish health care covers persons independently of their age.

The modified versions cover three domains: adolescents’ health behavior and responsibility, parental involvement, and adolescents’ overall transition readiness. The adolescent responsibility domain consists of nine items covering aspects related to taking blood samples, managing medication, renewing prescriptions, communication about the disease, communication with HCP, and medical appointment attendance. The parental involvement domain consists of the same nine aforementioned items. Adolescents’ responsibility and parental involvement are all rated on a 4-point Likert-type scale (*not at all responsible/involved, somewhat responsible/involved, mostly responsible/involved, completely responsible/involved*, and the added option, *not applicable*). A total mean score for adolescent’s responsibility and parental involvement was calculated if there were not more than four items missing per individual in each domain (a half-scale approach; range = 1-4). In the analyses, the additional option *not applicable* for each item is counted as a missing value.

Overall transition readiness includes two items: “Overall, how ready do you think you are to assume complete responsibility for your health care?” and “Overall, how ready do you think you are to transition from paediatric health care to adult health care?” The overall readiness mean scores are based on a 4-point Likert-type scale (*not at all ready, somewhat ready, mostly ready, completely ready*; range = 1-4).

#### Correlates

To determine which variables to include in the regression model, previous research describing and assessing transition readiness was reviewed. Therefore, as primary explanatory variables, the model includes disease-related knowledge, patient-related health, quality of life (QoL), and empowerment. Furthermore, illness perception, health behavior, and patient-specific characteristics have been added as potential explanatory factors (e.g., age, sex, and CHD complexity).

Sociodemographic information (age, sex, parental sex, and age) was collected along with the questionnaires. CHD complexity was obtained from the medical records and classified as mild, moderate, and complex according to Task Force 1 of the 32th Bethesda conference (Warnes et al., 2001).

Empowerment was measured using the Gothenburg Young Persons Empowerment Scale (GYPES-CHD). The GYPES-CHD consists of 15 items comprising five dimensions of empowerment: knowledge and understanding, personal control, identity, shared decision making, and enabling others. The total scores range from 15 to 75, with higher scores indicating a higher level of empowerment (Acuña Mora et al., 2018).

Disease-specific knowledge was measured with the Knowledge Scale for Adults With Congenital Heart Malformation (KnoCohm; Ronning, Arestedt, Erik Nielsen, Swahn, & Stromberg, 2013). The questionnaire comprises 19 items with four domains: general knowledge, medical treatment, endocarditis prophylaxis, and pregnancies and contraceptives. In the present study, only the domain of general knowledge is used to calculate a score. This domain includes a total of 11 items regarding knowledge about condition, treatment, endocarditis, physical activity, and heredity. A higher score (range 0-20) reflects higher level of knowledge.

QoL was measured using a vertical linear analogue scale (LAS) with a range from 1 to 100. QoL is defined as “the degree of overall life satisfaction that is positively or negatively influenced by individuals’ perception of certain aspects of life important to them, including matters both related and unrelated to health” (Moons et al., 2005, p. 299). Higher scoring implies a better perceived QoL (Moons et al., 2006).

Illness perception was measured with the Brief–Illness Perception Questionnaire (B-IPQ; Broadbent, Petrie, Main, & Weinman, 2006). The IPQ has eight items (0-10 points) covering cognitive and emotional illness perceptions, timeline, personal control, treatment control, identity of disease, illness coherence, emotional response, and concern. A higher total score (0-80) represents a more threatening view of the disease.

Patient-reported health was measured using the generic module of the Pediatric Quality of Life Inventory 4.0 (PedsQL 4.0; Petersen, Hagglof, Stenlund, & Bergstrom, 2009). The PedsQL generic module has 23 items in four domains (physical, emotional, social functioning, and school functioning) measured on a 5-point Likert-type scale (*never, almost never, sometimes, often*, and *almost always*). Higher total scores indicate better patient-reported health.

Health behavior was assessed with the Health Behavior Scale-CHD (HBS; Goossens et al., 2013). This instrument includes 15 items addressing behaviors related to physical activity, dental hygiene, and the use of alcohol, tobacco, and drugs. A total score is calculated (score = 0-100), and higher scores indicate unhealthier behaviors.

### Statistical Analysis

Statistical analyses were performed using IBM SPSS Statistics for Windows version 24 (IBM Corp.; Armonk, NY, USA). Descriptive statistics were expressed in absolute numbers, percentages, means, and standard deviations. Between-group comparisons were conducted using the Mann–Whitney U test for two groups and the Kruskal–Wallis test for comparison in more than two groups. For comparing dyads (paired tests), Wilcoxon rank test was used. To explore correlates of adolescents’ perceived overall readiness, adolescents’ responsibility, and parental involvement, univariable linear regression analyses (enter method) was performed, followed by multivariable analysis. The multivariable model was built by including variables with a *p* < .1 in the univariable analysis. The assumptions of linearity of residuals, absence of multicollinearity, and independence of observations were met by assessing Durbin–Watson statistics, variance inflation factor (VIF), and normal probability plots (P–P plots). All tests were two sided and the level of significance was established at *p* < .05.

For the analyses in the present article, we only included patients for whom data from the adolescent, the mother, and the father were available, that is, complete triads. To estimate the effect size, Cohen’s *d* was calculated (small effect size 0.2, medium effect size 0.5, and large effect size 0.8; Cohen, 1988).

## Results

From the 593 eligible patients, 202 (34%) participated in the study. After double checking the medical files, three patients were excluded. Of the 202 remaining patients, for 157 patients, data from both mothers and fathers were obtained, yielding complete triads. The mean age of the adolescents was 15.7 years, and 45.9% were female (Table 1). The study group and nonresponders significantly differed regarding age (15.7 ± 1.1 vs. 15.5 ± 1.1 years; *p* = .02, but the difference was clinically not meaningful (Cohen’s *d* = 0.18). There was no significant difference on CHD complexity and sex. The mean age of the mothers was 46.9 ± 4.9 years and of the fathers was 49.3 ± 5.5 years.

**Table 1.** Demographic Characteristics of the Participating Adolescents.

	*n* = (%)	*M* (±*SD*)
Age		15.7 (1.1)
Female	72 (45.9)	
Complexity of the CHD		
Mild	46 (29.3)	
Moderate	70 (44.6)	
Complex	41 (26.1)	
Education		
Elementary school	86 (57)	
Secondary school	63 (41.7)	
Other	2 (1.3)	
Living conditions		
Living with both parents	137 (87.8)	
Living alternately with mother or father	10 (6.4)	
Mother	3 (1.9)	
Father	3 (1.9)	
Other	3 (1.9)	
Taking any medication because of your heart condition?	36 (22.9)	
Born with another congenital condition?	24 (15.8)	
Needing treatment for any other condition?	24 (15.6)	

*Note.* CHD = congenital heart disease.

### The Level of Transition Readiness

The adolescents perceived a mean overall readiness of 2.7 ± 0.9, an adolescent responsibility score of 2.4 ± 0.7, and a parental involvement score of 3.6 ± 0.6 on a scale from 2 to 4 (Table 2). Adolescents reported a high level of responsibility for the items *taking medication as prescribed, explaining the medical condition to others, attending medical appointments*, and *communicating with the medical staff in person*. They scored lower on *scheduling appointments for specialist care* and for *calling in or ordering refills*. The adolescents reported the highest level of parental involvement for *getting monthly labs, scheduling primary care appointments*, and *communicating with medical staff over the phone* (Table 2). The parents’ assessments are also displayed in Table 2.

**Table 2.** Adolescents’, Mothers’, and Fathers’ Assessment of Overall Readiness, Adolescent Responsibility, and Parental Involvement.

Overall, how ready do you think you are to . . . Overall, how ready do you think your adolescent is to . . .	Overall readiness			Adolescent responsibility			Parental involvement		
	Adolescent *M* (±*SD*)	Mother *M* (±*SD*)	Father *M* (±*SD*)	Adolescent *M* (±*SD*)	Mother *M* (±*SD*)	Father *M* (±*SD*)	Adolescent *M* (±*SD*)	Mother *M* (±*SD*)	Father *M* (±*SD*)
Assume complete responsibility for your/their health care?	2.7 (0.9)	2.4 (1.0)	2.5 (1.0)						
Be transferred from care at a children’s hospital to adult care?	2.7 (1.0)	2.4 (1.0)	2.4 (1.0)						
Getting monthly labs				2.4 (1.3)	2.7 (1.3)	3 (1.2)	3.8 (0.8)	3.7 (0.7)	3.4 (0.9)
Taking medication as prescribed				3.4 (1.0)	3.4 (0.9)	3.3 (0.9)	3.0 (1.2)	3.1 (1.1)	2.9 (1.0)
Scheduling speciality care appointments				1.5 (1.0)	1.5 (1.0)	1.7 (1.1)	3.7 (0.8)	3.6 (0.8)	2.9 (1.2)
Scheduling primary care appointments				1.6 (1.0)	1.7 (1.1)	1.8 (1.1)	3.8 (0.6)	3.7 (0.7)	3.0 (1.1)
Calling in or ordering refills				1.5 (0.9)	1.5 (1.0)	1.4 (0.9)	3.7 (0.9)	3.6 (0.9)	2.7 (1.2)
Explaining medical condition to others				3.2 (0.9)	2.9 (1.0)	2.9 (1.0)	3.2 (1.0)	3.5 (0.8)	3.2 (0.9)
Attending medical appointments				3.2 (1.1)	3.3 (1.1)	3.3 (1.0)	3.7 (0.6)	3.7 (0.7)	3.4 (0.9)
Communicating with medical staff in person				3.2 (0.9)	3.4 (0.9)	3.4 (0.8)	3.6 (0.7)	3.7 (0.6)	3.4 (0.9)
Communicating with medical staff over the phone				1.7 (1.1)	1.8 (1.1)	1.9 (1.1)	3.8 (0.6)	3.7 (0.7)	3.1 (1.1)
Total *M* score (±*SD*)	2.7 (0.9)	2.4 (1.0)	2.4 (0.9)	2.4 (0.7)	2.5 (0.8)	2.6 (0.8)	3.6 (0.6)	3.6 (0.5)	3.2 (0.8)

**Figure 1.**
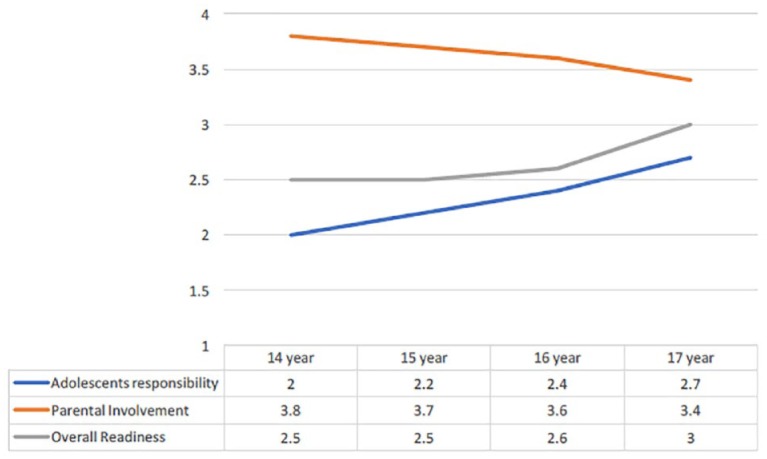
Adolescents’ perceived responsibility, parental involvement, and overall readiness for transition, per age cohort. *Note.* (*M*) Adolescents’ responsibility *p* = .001. Parental involvement *p* = .087. Overall readiness *p* = .021.

Univariable analyses demonstrated that higher age was significantly associated with increasing overall transition readiness (*p* = .021) and increasing adolescents’ responsibility (*p* = .001; Figure 1). The decreasing trend of parental involvement over the different age cohorts did not reach statistical significance (*p* = .087). None of the readiness domains was associated with sex or the complexity of the heart defect.

### Comparison of Transition Readiness Between Adolescents and Mothers or Fathers

On overall readiness, the adolescents (2.7 ± 0.9) scored higher than their mothers (2.4 ± 1, *p* = .001; Cohen’s *d* = 0.32) and fathers (2.4 ± 0.9, *p* = .001; Cohen’s *d* = 0.33; Table 2). No significant difference was seen in the adolescents’ reported responsibility (2.4 ± 0.7) versus their mothers’ (2.5 ± 0.8, *p* = .583) or fathers’ (2.6 ± 0.8, *p* = .119). Adolescents scored significantly higher on parental involvement (3.6 ± 0.6) compared with their fathers (3.2 ± 0.8, *p* =.001; Cohen’s *d* = 0.56), but no difference was seen between the adolescents and their mothers (3.6 ± 0.5, *p* = .811; see Table 2).

### Potential Correlates of Transition Readiness in Adolescents With CHD

The results from the multivariable analyses showed that higher levels of adolescents’ overall readiness were associated with lower IPQ score (less threatening view; β = –.142, *p* = .047), a higher level of empowerment (β = .146, *p* = .026), and with higher mothers’ and fathers’ overall readiness scores (mothers β = .277, *p* = .002; fathers β = .281, *p* = .001; Table 3). A higher level of adolescents’ responsibility scores was associated with higher age (β = .168, *p* = .039) and higher mothers’ and fathers’ adolescent responsibility scores (mothers β = .367, *p* = .003; fathers β = .29, *p* = .02). The adolescents’ parental involvement scores were negatively associated with adolescents’ age (β = –.212, *p* = .025) and positively with the mothers’ parental involvement scores (β = .215, *p* = .023; Table 3).

**Table 3.** Correlates of Adolescents’ Perceived Overall Readiness, Adolescent’s Responsibility, and Parental Involvement.

Correlates	Overall readiness		Adolescent responsibility		Parental involvement	
	*B* (*SE*)	β	*B* (*SE*)	β	*B* (*SE*)	β
Sex	.193 (0.113) *p* = .90	.109	—	—	—	—
Age	.099 (0.051) *p* = .055	.127	.110 (0.052) *p* = .039	.168	−.117 (0.051) *p* = .025	−.212
QoL	−.002 (0.003) *p* = .599	−.037	—	—	—	—
Illness Perception Total score	−.011 (0.005) *p* = .047	−.142	—	—	—	—
GYPES	.020 (0.009) *p* = .026	.146	.012 (0.008) *p* = .150	.126	—	—
PedsQL Total score	.006 (0.005) *p* = .257	.093	—	—	—	—
Health Behavior Scale	−.006 (0.006) *p* = .324	−.064	−.005 (0.006) *p* = .423	−.062	—	—
M_Overall Readiness	.255 (0.080) *p* = .002	.277	—	—	—	—
F_Overall Readiness	.261 (0.080) *p* = .001	.281	—	—	—	—
M_Adolescent’s Readiness	—	—	.336 (0.109) *p* = .003	.367	—	—
F_Adolescent’s Readiness	—	—	.259 (0.109) *p* = .02	.290	—	—
M_Parental Involvement	—	—	—	—	.242 (0.105) *p* = .023	.215
F_Parental Involvement	—	—	—	—	—	—
	*R*^2^ = .504		*R*^2^ = .549		*R*^2^ = .085	

*Note.* QoL = quality of life; GYPES = Gothenburg Young Persons Empowerment Scale.

## Discussion

The present cross-sectional study investigated transition readiness among Swedish adolescents with CHD and potential factors associated with transition readiness. The adolescents’ overall readiness score was moderate. The perceived transition readiness and adolescent’s responsibility increased with age of the young person. Comparable results have been described in previous, similarly designed studies on adolescents with chronic disease (e.g., Eaton et al., 2017; Uzark et al., 2015; van Staa et al., 2011). One argument is that the results are most likely associated with the developmental process of going from adolescence to adulthood. The increased maturity and life skills developed throughout this process increase the adolescents’ involvement in their care and their sense of readiness for transition (Christie & Viner, 2005).

The adolescents’ perception of overall readiness differed from their parents’ reports, with the adolescents perceiving themselves as more ready to take over responsibility for their health and to be transferred to adult care than the parents reported. This is in line with a previous report of Sawicki, Kelemen, and Weitzman (2014), who studied adolescents with chronic conditions. Such results could indicate that the adolescents overestimate their capacity. Another explanation could be that the parents do not acknowledge the adolescents’ actual skills, underestimating their capacity. However, in the present study, parents’ overall transition readiness perception was associated with an increased adolescent’s overall readiness score after adjusting for other factors, perhaps suggesting that adolescents might feel encouraged when parents trust in their ability to take charge of their life. A third explanation could be that even if parents underestimate their adolescent’s abilities or adolescents overestimate their skills, there is still a clear association between the scores. However, in contrast to Sawicki and colleagues (2014), our results did not reveal any difference in adolescents’ responsibility when comparing the mothers’ and fathers’ assessments with the adolescent. There was a high level of parental involvement reported among the adolescents and their parents. However, fathers reported significantly lower levels. Although the adolescents scored highly on, for example, explaining their condition to others, they still reported high parental involvement. High parental involvement is suggested to be a barrier to independence and self-efficacy (Helgeson et al., 2008). Mackie et al. (2016) reported a high level of parental overprotection perceived by adolescents during transition. However, the result of this study could not establish whether the level of parental involvement predicts overprotection or whether it is one dimension of parenting an adolescent with a chronic condition. A shift in roles is crucial and parental involvement in the adolescents’ care is an important factor in the process of achieving independence (Bratt et al., 2018). However, prior research indicated that the adolescents still want to rely on their parents in case of questions or uncertainties (Allen, Channon, Lowes, Atwell, & Lane, 2011; Moons et al., 2009). Indeed, it is important to bear in mind the parents’ expertise, giving them the opportunity to transfer their knowledge and experiences to the adolescent.

The levels of interdependencies and the vulnerabilities that may accompany incongruent levels of collaboration within the dyad/triad are important aspects to be aware of (Lyons & Lee, 2018). In a clinical setting, the collaboration within the dyads/triads is crucial to acknowledge in meeting the needs of the individual adolescent and the parents. It can be questioned whether independence is the ultimate goal or rather the concordant interdependency. A discussion of redefinition of roles is required. Furthermore, the increasing support from peers and partners should also be taken into consideration.

The results in the present study support current understandings about age as one explanatory factor for increased overall readiness and adolescents’ responsibility. Moreover, the level of parental involvement and adolescents’ level of empowerment are factors that should be considered. The results support the idea that empowerment is closely associated to transition readiness. This emphasizes how important it is for nurses to strengthen the adolescent’s responsibility in health management, involvement, and self-care, and to support them in being involved and knowledgeable about their own health condition and care.

A less threatening view of the illness, including cognitive and emotional illness perceptions such as personal control, treatment control, identity of disease, illness coherence, emotional response, and concerns (B-IPQ), was associated with an increased overall readiness in the adolescents. It is conceivable that increased awareness about the condition leads to increased self-confidence and a sense of security in handling their disease. Reid et al. (2004) reported similar findings: Better transition readiness was associated with awareness of the risk of complications or adverse effects if cardiac follow-up was neglected. Self-efficacy in attending cardiac appointments was also associated with better transition readiness.

The complexity of the disease, gender, health status, QoL, disease-specific knowledge, or health behaviors were not associated with transition readiness. This suggests that these factors may be less important for transition readiness. However, this is not congruent with previous studies in which transition readiness has been reported to be associated with QoL (Uzark et al., 2015) and condition-specific knowledge (Stewart et al., 2017).

Transition readiness questionnaires could play an important role in clinical practice to determine timing of transfer to adult care, in that, they consider other factors in addition to chronological age. Further exploration is nevertheless needed to determine cutoffs indicating when the adolescent appears to be ready to transfer. Measurements such as the RTQ can contribute important information but should be seen as one of several factors to consider when assessing transition readiness.

Some methodological considerations need to be addressed in this study. First, SWEDCON (SWEDCON, 2016) was used to identify patients fulfilling the inclusion criteria at four university hospitals. However, despite the use of a modified Dillman approach, the response rate was only fair (Dillman, 1983). The results might, therefore, disproportionately reflect the CHD population, which hampers the generalizability of the results. We considered it important to invite all adolescents with CHD, regardless of complexity of the disease, as adolescents with less complex conditions also need continuity of care and are assumed to transfer care upon reaching adulthood (Andemariam et al., 2014; Gurvitz et al., 2013). The CHD population in this study had wide-ranging complexity of disease and medical needs.

Second, many adolescents have additional congenital conditions and need treatment for other conditions. These aspects have not been taken into consideration when analyzing the data in the current study.

Third, the RTQ is not a widely used transition readiness instrument but has the benefit of having a parental proxy version. The RTQ was originally developed for adolescents with kidney transplants, and the domain for adolescents’ responsibility and parental involvement contains several items about medication and labs. In the present study, only 23% of the study population were taking daily medication and the majority did not need monthly labs. This led to a high number of responders choosing the option *not applicable* for items related to medication and monthly labs. Fourth, the cross-sectional design does not help to determine cause and effect relationships. Fifth, we have not corrected for multiple testing because we consider this study rather to be explorative.

### Implications for Family-Focused Practice With Families

The International Family Nursing Association (IFNA) has developed a Position Statement of Generalist Competencies for Family Nursing Practice with an emphasis on families’ strengths;, the support of family and individual growth, the improvement of family self-management abilities, facilitation of successful life transitions, the improvement and management of health, and the mobilization of family resources (IFNA, 2015).

By assessing transition readiness, care providers can promote the care of adolescents with chronic conditions *and* their parents. The assessment of transition readiness is one of several essential aspects of care as HCPs support adolescents during the development of autonomy in the self-management of a chronic condition. The present study provides evidence that parents also need to be included in the adolescent’s health care transition. At the same time, HCPs need to assess the level of parental involvement to determine whether it enhances or thwarts attainment of the adolescent’s development of health care autonomy. The present study underscores the importance of a family-oriented clinical setting.

## Conclusion

The present study supports the notion that adolescents’ responsibility and overall readiness elevate with age. Nevertheless, age in itself does not solely determine when the adolescent is ready for transfer. Because empowerment and illness perception are also related to transition readiness, emphasis should be given to empowering adolescents and to supporting them in gaining personal control, increased awareness about their condition, and achieving self-management. Although adolescents’ and parents’ perceptions differed, they still correlate and show that higher scoring by parents is associated with higher self-perceived readiness in adolescents. The results of this study confirm the significance of the adolescents and parents multifaceted perspective during the adolescent’s transition into adulthood. By using a triadic evaluation, the results from the present study significantly extend what is currently known about this population. Future nursing intervention must take associated factors and the change in interdependency within the triads into consideration.
